# 20 Years of Real-World Data to Estimate the Prevalence of Heart Failure and Its Subtypes in an Unselected Population of Integrated Care Units

**DOI:** 10.3390/jcdd9050149

**Published:** 2022-05-07

**Authors:** Cristina Gavina, Daniel Seabra Carvalho, Filipa Valente, Filipa Bernardo, Ricardo Jorge Dinis-Oliveira, Carla Santos-Araújo, Tiago Taveira-Gomes

**Affiliations:** 1 Cardiology Department, Pedro Hispano Hospital, Senhora da Hora, 4464-513 Matosinhos, Portugal; cgavina@med.up.pt (C.G.); up202103237@edu.med.up.pt (D.S.C.); 2 Medical Department, Evidence Generation, AstraZeneca, 2730-097 Barcarena, Portugal; filipa.valente@astrazeneca.com (F.V.); filipa.bernardo@astrazeneca.com (F.B.); 3 Department of Public Health and Forensic Sciences, and Medical Education, Faculty of Medicine, University of Porto, 4200-319 Porto, Portugal; 4 TOXRUN—Toxicology Research Unit, University Institute of Health Sciences, Advanced Polytechnic and University Cooperative (CESPU), CRL, 4585-116 Gandra, Portugal; tiagogomes@med.up.pt; 5 UCIBIO-REQUIMTE, Laboratory of Toxicology, Department of Biological Sciences, Faculty of Pharmacy, University of Porto, 4050-313 Porto, Portugal; 6 MTG Research and Development Lab, 4200-604 Porto, Portugal; 7 Nephrology Department, Pedro Hispano Hospital, Senhora da Hora, 4464-513 Matosinhos, Portugal; csaraujo@med.up.pt; 8 UnIC@RISE, Department of Surgery and Physiology, Faculty of Medicine, University of Porto, 4200-319 Porto, Portugal; 9 Department of Community Medicine, Information and Decision in Health, Faculty of Medicine, University of Porto, 4050-313 Porto, Portugal; 10 Center for Health Technology and Services Research (CINTESIS), 4200-450 Porto, Portugal; 11 Faculty of Health Sciences, University Fernando Pessoa (FCS-UFP), 4249-004 Porto, Portugal

**Keywords:** heart failure, type 2 diabetes mellitus, prevalence, integrated care users, chronic kidney disease

## Abstract

Introduction: Heart failure (HF) is a clinical syndrome caused by structural and functional cardiac abnormalities resulting in the impairment of cardiac function, entailing significant mortality. The prevalence of HF has reached epidemic proportions in the last few decades, mainly in the elderly, but recent evidence suggests that its epidemiology may be changing. Objective: Our objective was to estimate the prevalence of HF and its subtypes, and to characterize HF in a population of integrated care users. Material and Methods: A non-interventional cross-sectional study was performed in a healthcare center that provides primary, secondary and tertiary health cares. Echocardiographic parameters (left ventricle ejection fraction (LVEF) and evidence of structural heart disease) and elevated levels of natriuretic peptides were used to define two HF phenotypes: (i) HF with a reduced ejection fraction (HFrEF, LVEF ≤ 40% and either NT-proBNP ≥ 400 pg/mL (≥600 pg/mL if atrial fibrillation (AF)/flutter) or BNP ≥ 100 pg/mL (≥125 pg/mL if AF/flutter)) and (ii) HF with a non-reduced ejection fraction (HFnrEF), which encompasses both HFpEF (LVEF ≥ 50% and either NT-proBNP ≥ 200 pg/mL (≥600 pg/mL if AF/flutter) or BNP ≥ 100 pg/mL (≥125 pg/mL if AF/flutter) in the presence of at least one structural cardiac abnormality) and HF with a mildly reduced fraction (HFmrEF, LVEF within 40–50% and either NT-proBNP ≥ 200 pg/mL (≥600 pg/mL if AF/flutter) or BNP ≥ 100 pg/mL (≥125 pg/mL if AF/flutter) in the presence of at least one structural cardiac abnormality). The significance threshold was set at *p* ≤ 0.001. Results: We analyzed 126,636 patients with a mean age of 52.2 (SD = 18.3) years, with 57% (*n* = 72,290) being female. The prevalence of HF was 2.1% (*n* = 2700). The HF patients’ mean age was 74.0 (SD = 12.1) years, and 51.6% (*n =* 1394) were female. Regarding HF subtypes, HFpEF accounted for 65.4% (*n* = 1765); 16.1% (*n* = 434) had HFmrEF and 16.3% (*n* = 439) had HFrEF. The patients with HFrEF were younger (*p <* 0.001) and had a history of myocardial infarction more frequently (*p <* 0.001) compared to HFnrEF, with no other significant differences between the HF groups. The HFrEF patients were more frequently prescribed CV medications than HFnrEF patients. Type 2 Diabetes Mellitus (T2D) was present in 44.7% (*n* = 1207) of the HF patients. CKD was more frequently present in T2D vs. non-T2D HF patients at every stage (*p <* 0.001), as well as stroke, peripheral artery disease, and microvascular disease (*p <* 0.001). Conclusions: In this cohort, considering a contemporary definition, the prevalence of HF was 2.1%. HFrEF accounted for 16.3% of the cases, with a similar clinical–epidemiological profile having been previously reported in the literature. Our study revealed a high prevalence of patients with HFpEF (65.4%), raising awareness for the increasing prevalence of this entity in cardiology practice. These results may guide local and national health policies and strategies for HF diagnosis and management.

## 1. Introduction

Heart failure (HF) is a clinical syndrome caused by structural and functional cardiac abnormalities, resulting in the impairment of ventricular filling and/or the ejection of blood. HF results in significant morbidity and mortality, with a 1-year mortality rate of 7.2% and a 1-year hospitalization rate of 31.9% in patients with chronic HF [1]. In developed countries, HF is estimated to affect about 2% of the adult population, with an annual incidence of 5–10/1000 persons [2]. In Portugal, the EPICA study estimated the prevalence of HF in adults to be about 4.4%, reaching 12.7% in septuagenarians, and 16.1% in patients over 80 years old [3].

HF is described as an age-dependent disease, with its global incidence being lower in women than in men [4]. In both genders, the incidence of HF is low before the age of 60 years, increasing significantly afterwards. The incidence of HF is higher in women than men at the age of 85 years, as HF and ageing are competing risks for death, and men’s life expectancy is lower [5]. A recent analysis predicted that the number of HF patients in Portugal will continue to rise, estimating a relative increase of 30% in prevalence by 2035, and 33% by 2060, comparatively to 2011 [2]. Similarly, a study conducted in the United Kingdom showed that the absolute number of people living with HF increased by 23% from 2002 to 2014 [6]. This steep increase parallels the estimated population ageing in the same period [7].

Cardiovascular risk factors such as obesity, hypertension, diabetes, smoking, coronary artery disease and a history of stroke can significantly increase the risk of developing HF. A single factor such as Type 2 Diabetes Mellitus (T2D) increases the risk of developing HF up to two-fold in men and five-fold in women, and negatively impacts prognosis [5,8,9]. Moreover, the prevalence of T2D among patients with HF ranges between 25 and 40% in registries [10], and 30 and 50% in HF clinical trials [11,12,13,14,15,16,17].

HF is usually subclassified into three subtypes according to the left ventricular ejection fraction (LVEF): (i) HF with a preserved ejection fraction (HFpEF, LVEF ≥ 50%), (ii) HF with a mildly reduced fraction (HFmrEF, LVEF] 40–50 (%), and (iii) HF with a reduced ejection fraction (HFrEF, LVEF ≤ 40%) [1]. This classification is now considered the cornerstone for HF diagnosis and characterization according to the 2021 European Society of Cardiology (ESC) and the 2022 AHA/ACC/HFSA HF guidelines [18,19]. In particular, the reported prevalence of HFpEF is increasing, likely due to clinicians’ awareness, refined echocardiographic and biomarker characterization, the increased burden of ageing, and lifestyle-related risk factors such as obesity and diabetes. HFpEF accounts for about half of the hospitalizations for HF [20], and for a larger proportion (51–63%) of HF cases in the community [21,22,23]. Even though the morbidity and mortality rates for HFpEF are lower than those for HFrEF, the commonly associated cardiovascular and non-cardiovascular comorbidities have an unfavorable impact on its evolution [24]. Patients with HFpEF are older, more likely to have hypertension and atrial fibrillation [25] (AF), and around 45% have T2D [9].

In order to improve our understanding of HF patients and the associated risk factors—particularly T2D—a study was conducted in a Portuguese center to estimate the prevalence of HF and its subtypes, and to characterize HF patients’ demography and clinical situation. Such an understanding is paramount for the improvement of health policies and strategies concerning HF diagnosis and management.

## 2. Materials and Methods

### 2.1. Study Settings

This was a non-interventional cross-sectional study performed in the Health Local Unit of Matosinhos (Unidade Local de Saúde de Matosinhos, ULSM), a regional health system in the district of Matosinhos in the north of Portugal, englobing 14 Primary Care Health Units (PCHUs) assisted by the same Secondary and Tertiary Care Health Unit (STCHU)-Pedro Hispano Hospital. We selected all persons aged 18-years-old or more who attended healthcare units at least once in the 3 years before the index date. We analyzed the health records of all persons older than 18 years who were alive at the time of data access and had attended at least one medical appointment at any ULSM health unit within the past 3 years. Data access for the analysis was granted after approval by the Ethical Committee and Data Protection Officer of the ULSM (translated from Comissão de Ética para a Saúde da Unidade Local de Saúde de Matosinhos), approval codes N.º34/CE/JAS of 23-04-2020 (original) and N.º64/CE/JAS of 10-07-2020 (addenda). De-identified data were extracted from electronic heath records according to the HIPAA Safe Harbor Standard; data regarding age, gender, and comorbidities were classified by the International Classification of Diseases (ICD)-9 and -10 codes, and medications were registered according to the Anatomical Therapeutic Chemical Classification System.

### 2.2. Study Definitions

#### 2.2.1. Type 2 Diabetes Mellitus Definition

T2D was defined as the presence of at least one measurement of HbA1c ≥ 6.5% or occasional plasma glucose ≥ 200mg/dL.

#### 2.2.2. Heart Failure Definition

HF diagnosis was derived by adapting criteria used in three HF clinical the trials DAPA-HF [14], PARADIGM-HF [26] and PARAGON-HF [13], and the guidelines for the diagnosis and treatment of HF from the 2021 ESC guidelines [18]. We used echocardiographic parameters (LVEF and evidence of structural cardiac disease–left atrial volume (LAV), indexed LAV, left atrial diameter (LAD), interventricular septum thickness (IVS), posterior wall thickness (PWL), left ventricular mass (LVM) and indexed LVM), an E/e’ ratio at rest >9, and laboratory measurements of B-type natriuretic peptide (BNP) and NT-proBNP to stratify HF. In the cohort, HF was not actively screened for echocardiographic parameters and natriuretic peptides, as these measurements are not widely available in primary care. The results for the HF subtypes were reported in two groups: (i) HFrEF patients, and (ii) HFnrEF that encompasses both HFpEF and HFmrEF patients. For 2.3% (*n* = 68) of the HF patients it was not possible to adequately stratified them into any of these groups, and they were excluded.

#### 2.2.3. Heart Failure with Reduced LVEF Definition

HFrEF was defined as LVEF ≤ 40% and either NT-proBNP ≥ 400 pg/mL (≥600 pg/mL if AF/flutter) or BNP ≥ 100 pg/mL (≥125 pg/mL if AF/flutter).

#### 2.2.4. Heart Failure with Mildly Reduced LVEF Definition

HFmrEF was defined as LVEF within 40 and 50% and either NT-proBNP ≥ 200 pg/mL (≥600 pg/mL if AF/flutter) or BNP ≥ 100pg/mL (≥125 pg/mL if AF/flutter) in the presence of at least one structural cardiac abnormality (indexed LAV > 34 mL/m^2^, LAV > 50 mL, LAD > 38mm, IVS > 11 mm, PW > 11 mm, E/e’ ratio at rest >9, indexed LVM > 115 g/m^2^ for males, indexed LVM > 95 g/m^2^ for women).

#### 2.2.5. Heart Failure with Preserved LVEF Definition

HFpEF was defined as LVEF ≥ 50% and either NT-proBNP ≥ 200 pg/mL (≥600 pg/mL if AF/flutter) or BNP ≥ 100 pg/mL (≥125 pg/mL if AF/flutter) in the presence of at least one structural cardiac abnormality (indexed LAV > 34 mL/m^2^, LAV > 50 mL, LAD > 38 mm, IVS > 11 mm, PW > 11 mm, E/e’ ratio at rest > 9, indexed LVM >115 g/m^2^ for males, indexed LVM > 95 g/m^2^ for women).

#### 2.2.6. Additional Comorbidities Definition

Myocardial infarction, hypertension, AF, stroke, peripheral artery disease and microvascular disease were defined by the presence of at least one ICPC-2, ICD-9 or ICD-10 code. Plasmatic creatinine determination was performed in the same laboratory, and was used for the estimated glomerular filtration rate (eGFR) calculation. Chronic Kidney Disease (CKD) was defined as having at least one measurement of eGFR (<60 mL/min/1.73 m^2^). CKD was staged using eGFR calculated using the Modification of Diet in Renal Disease (MDRD) formula [27,28]. eGFR and indexed echocardiographic parameters were computed from scratch using existing lab and measurement data.

#### 2.2.7. Medication Definitions

Patients were considered to undergo a given medication if there was at least one recorded prescription including that medication within 365 days prior to the index date. The medications were defined from national prescription codes and mapped to ATC codes.

### 2.3. Statistical Analysis

The patient characteristics were reported using mean and standard deviation (SD), median and interquartile range (IQR), and absolute and relative frequencies, as appropriate. Univariate differences between subgroups of interest were assessed using a non-paired T-Student test for age, a Mann–Whitney U test for every other continuous variable, and a Chi-squared test for categorical variables. The significance threshold was adjusted using Bonferroni correction and was set at *p* ≤ 0.001. The statistical analysis was performed using Apache Spark Framework version 2.4526 [29] and R version 4.0327 [30].

## 3. Results

A total of 126,636 users (mostly Caucasian) matching the inclusion criteria were enrolled, representing approximately 90% of the population of the geographic region of Matosinhos according to the 2021 Portuguese Census (the eighth most inhabited municipality in the country and the fourth in the northern region). The patients’ mean age was 52.2 (SD = 18.3) years, and 57% (*n* = 72,290) were female. The prevalence of HF was 2.13% (*n* = 2700). The prevalence of HF was 2.20% (*n* = 2591) in patients over 50 years, 5.23% (*n* = 831) in septuagenarians, and 10.88% (*n* = 1006) in patients over 80 years. In patients less than 50 years old, the prevalence of HF was residual (0.8%; *n* = 109).

### 3.1. Characterization of Patients with HF

**Figure 1:**
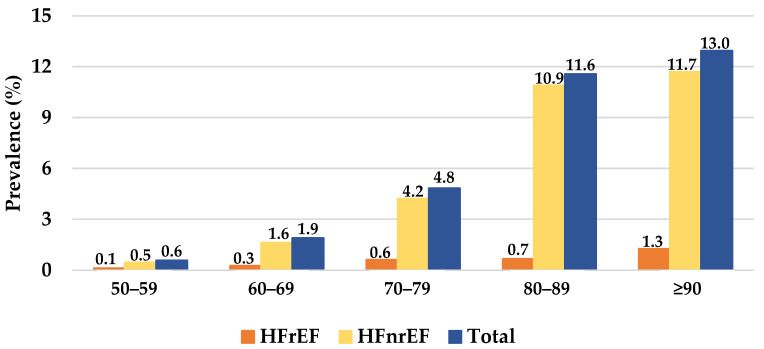
The prevalence of heart failure in females per heart failure subtype and age group. HFrEF, heart failure with a reduced ejection fraction; HFnrEF, heart failure with a non-reduced ejection fraction.

**Figure 2:**
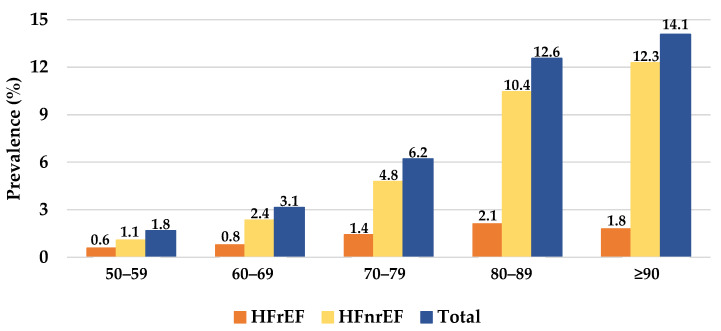
The prevalence of heart failure in males per heart failure subtype and age group. HFrEF, heart failure with a reduced ejection fraction; HFnrEF, heart failure with a non-reduced ejection fraction.

Detailed results regarding demographics, comorbidities, cardiovascular medications, clinical assessment, and echocardiogram results of heart failure patients are presented in Table 1. The HF patients’ mean age was 74.0 (SD = 12.1) years, and 51.6% (*n* = 1394) were female. HFpEF accounted for 65.4% (*n* = 1765) of the HF patients. HFmrEF accounted for 16.1% (*n* = 434) and HFrEF accounted for 16.3% (*n* = 439) of the HF patients. The prevalence of HF increased with age both in female and male patients (Figure 1 and Figure 2). AF was present in 41.1% (*n* = 1110) of patients, and myocardial infarction was present in 31.0% (*n* = 836) of patients. T2D was present in 44.7% (*n* = 1207) of the HF patients. CKD was present in 66.8% of HF patients, and 7.4% (*n* = 201) had an eGFR ≤ 15 mL/min. The majority the HF patients were under cardiovascular medication. Statins were used in 64.7% (*n* = 1747), hypertension medication was used in 60% (*n* = 1619), beta blockers were used in 60% (*n* = 1620), and diuretics were used in 46.9% (*n* = 1266) of the patients. No cardiovascular medications were recorded in 13.1% (*n* = 353) of the patients.

### 3.2. Characterization of HF Subtypes

HFrEF accounted for 18.6% (*n* = 439) and HFnrEF accounted for 81.4% (*n* = 2199) of theHF patients. The mean age for the HFrEF patients was 70.0 years old (SD = 12.0) vs. 74.7 years old (SD = 12.0) for the HFnrEF patients (*p* < 0.001). Females accounted for 33.9% (*n* = 139) of HFrEF patients vs. 55.0% (*n* = 1209) of HFnrEF patients (*p* < 0.001). Detailed results are shown in Table 2. A history of myocardial infarction was present in 45.6% (*n* = 200) of HFrEF patients vs. 28.4% (*n* = 624) of HFnrEF patients (*p* < 0.001). There were no other significant differences in comorbidities between the HF groups. The HFrEF patients were under cardiovascular medications more frequently than the HFnrEF patients (Table 2), namely antiplatelet agents, angiotensin-converting enzyme inhibitors (55.6% vs. 32.7%, *p* < 0.001), beta blockers (79.3% vs. 55.8%, *p* < 0.001), and aldosterone receptor antagonists (48.7% vs. 8.3%, *p* < 0.001).

### 3.3. Characterization of the Patients with HF and T2D

**Figure 3:**
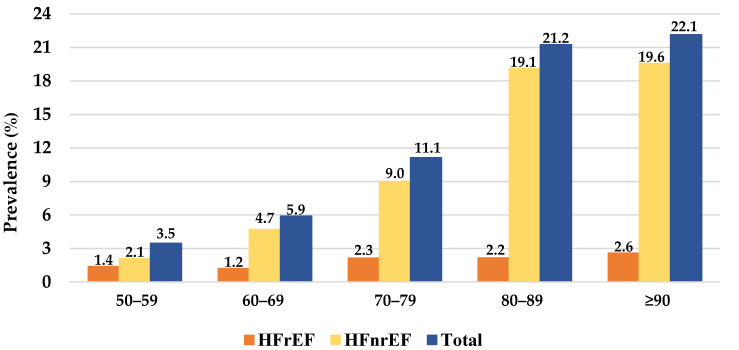
The prevalence of heart failure in Type 2 Diabetes Mellitus patients per heart failure subtype and age group. HFrEF, heart failure with a reduced ejection fraction; HFnrEF, heart failure with a non-reduced ejection fraction.

Detailed clinical assessment, laboratory test and echocardiogram results, and diabetes medication of patients with heart failure and type 2 Diabetes Mellitus are provided in Table 3. T2D was present in 44.7% (*n* = 1207) patients. T2D patients had mean age of 75.3 years (SD = 75.2) while patients without T2D had mean age of 72.3 years (SD = 13.4) (*p* < 0.001). Females represented 51.5% of the T2D patients and 52.0% of the non-T2D patients (*p* = 0.912). The median T2D duration was 3.0 years (IQR = 6.0). Figure 3 describes the prevalence of HF for T2D patients per HF subtype and age group. CKD was more frequently present in T2D vs. non-T2D patients at every stage (*p* < 0.001). Stroke occurred in 32.7% (*n* = 395, *p* < 0.001), peripheral artery disease occurred in 9.9% (*n* = 119, *p* < 0.001) and microvascular disease occurred in 35.4% (*n* = 427, *p* < 0.001) of the T2D patients (Table 4). Most of the T2D patients were under cardiovascular medication. Statins were used in 69.6% (*n* = 840), beta blockers were used in 63.5% (*n* = 767), and hypertension medications were used in 62.7% (*n* = 757) of the T2D patients. No cardiovascular medication was recorded in 9.5% (*n* = 115) of the T2D patients.

The majority of T2D patients were under diabetes medications. Metformin was used in 48.5% (*n* = 585) and DPP-4 inhibitors were used in 41.5% (*n* = 501) of the T2D patients. No diabetes medication was recorded in 28.4% (*n* = 343) of the T2D patients.

## 4. Discussion

HF is a clinical multisystemic syndrome defined by specific symptoms and signs due to structural and/or functional heart abnormalities, which lead to inadequate cardiac output and/or increased intraventricular filling pressure [31]. In this study, we aimed to assess the prevalence of HF and its subtypes in a Portuguese center, as well as associated risk factors, such as T2D. The prevalence of HF estimated in this study appears to be in line with other estimates reported in the literature for the adult population in developed countries. Nevertheless, compared with the EPICA study performed more than 20 years ago [3], which also assessed the prevalence of HF in the adult Portuguese population, the estimated prevalence in our study was nearly half (2.13% versus 4.36%) of that which was previously reported. Patient sampling, case definition and diagnostic criteria differences might explain this variation. Indeed, in the EPICA study, the study sample consisted of patients registered in their local primary healthcare centers, as well as those who were institutionalized. Moreover, the HF case definition included symptoms of exercise intolerance, signs of fluid retention (using the Boston questionnaire), the use of HF medications (with diuretics in monotherapy or in association with angiotensin-converting enzyme inhibitors, digitalis, or hydralazine plus nitrates), as well as evidence of cardiac dysfunction in an echocardiogram. Additionally, in the EPICA study, more objective data such as the natriuretic peptide levels were not yet available, and echocardiographic evaluation did not consider the ejection fraction or left atrial volume. All of these data are now considered the cornerstone for HF diagnosis and characterization according to the 2021 ESC and the 2022 AHA/ACC/HFSA HF guidelines [18,19]. The discrepant criteria for HF assessment might have contributed to a broader definition of HF in EPICA, and might thus explain the higher prevalence in comparison with other studies from European countries [32,33]. In our work, we considered individuals attending a regional health system in the district of Matosinhos in the north of Portugal, englobing 14 PCHU assisted by the same STCHU-Pedro Hispano Hospital. We did not assess clinical information regarding signs or symptoms of fluid retention; thus, we relied on natriuretic peptide measurements as a surrogate for the clinical suspicion of HF. Our definition of HF attempted to respect the current understanding of the disease, by combining natriuretic peptide measurements and structural cardiac changes with cut-offs used in recent HF trials such as DAPA-HF [14], PARADIGM-HF [26] and PARAGON-HF [13]. This approach certainly helped to increase the specificity for diagnosis, and is comparable to that reported in other countries [32,33].

Despite variations in diagnostic criteria, several relevant studies estimated that over half of all heart failures have a preserved ejection fraction, and the ratio of HFpEF to HFrEF is increasing [34,35]. Our study also revealed a high prevalence of patients with HFpEF among those diagnosed with HF (65.4%). Moreover, according to community-based studies, approximately 50% of patients with HF have HFpEF [36], but these estimates have been increasing over time, and may be underestimated. Indeed, population ageing and the increasing prevalence of cardiovascular risk factors, such as hypertension and T2D, both commonly associated with HFpEF, may partially explain our results. In addition, previous reports [37] emphasized that it is likely that HFpEF cases are easily confused with other entities that are symptomatically similar. This may lead to a misclassification of the records and, therefore, an underestimation of the real prevalence of this prognostically severe syndrome. As such, the very high percentage of patients with HFpEF in our cohort highlights this fact, probably more closely reflects the real prevalence, and represents a clear awareness that may impact future resource allocation and health policies. In addition, while the current guidelines [18] give a large space to HFrEF treatment, only few indications (e.g., the use of diuretics to reduce the signs and symptoms of congestion) are given for that of HFpEF and HFmrEF. Recent evidence points to the beneficial effect of SGLT2i in the reduction of the risk of major HF outcomes in patients with HFpEF [38,39].

In comparison to the HFnrEF subgroup, the patients with HFrEF were younger, often male, and commonly had a history of myocardial infarction. This profile is in line with prior literature [40]. Furthermore, the history of myocardial infarction likely explains the significantly higher rate of antiplatelet drugs and statins among patients with HFrEF compared to those with HFnrEF. It is noteworthy that there are few community studies evaluating the prevalence of HFpEF, and none reporting this statistical figure for HFmrEF. Moreover, as no specific information on symptoms or physical examination were available, conservative natriuretic peptide cut-off levels were selected, as well as pre-defined echocardiography criteria, respecting the most recent recommendations for HF diagnosis [18].

The patients with HFrEF had significantly higher prescription rates of prognostic-modifying drugs, such as angiotensin-converting enzyme inhibitors, beta blockers, and aldosterone receptor antagonists. These findings may be related to the higher disease burden, and may reflect the guidance provided for multi-target treatment in order to promote neurohormonal blockade in HF, which is a key element to counteract adverse cardiac remodeling [18].

T2D was present in 44.7% of the HF patients. While HF may play a causal role in the development of T2D, T2D is in turn a powerful risk factor for the development of HF [21]. Other studies estimate T2D to be present in 10 to 30% of HFrEF patients [41], and in 45% of HFpEF patients [9]. Our results show that HF patients with T2D have a higher burden of cardiovascular disease compared to non-T2D patients, in particular CKD. This fact highlights the reciprocal cardio–renal relationship, which extends from pathophysiological mechanisms to therapeutic implications [42]. Most patients with HF and T2D were being treated with antidiabetic agents, mainly metformin (48.5%). In recent cardiovascular outcome trials (CVOT), sodium–glucose cotransporter-2 (SGLT-2) inhibitors demonstrated robust results in the reduction of cardiovascular events and hospitalizations for HF [43,44]; currently, along with metformin, SGLT-2 inhibitors are indicated as the first-line pharmacological approach for diabetes in heart failure [45]. This was not observed in our population, in which only 9% of the T2D patients were under treatment with SGLT-2 inhibitors, and may be explained by the recency of such recommendations, and by the presence of renal function below the label threshold.

This study has some limitations. ULSM serves a predominantly urban population with broad primary healthcare coverage, and thus may not be representative of other regions of Portugal. The analysis performed was based on available data in electronic health records and an arbitrary definition of diseases; thus, the results cannot be directly compared with epidemiological studies designed for prevalence estimation. Patients were considered to undergo a given medication if there was at least one recorded prescription within 365 days prior to the index date. This definition may have caused our results to be underestimated, as patients may have medications filled for a longer period, may have filled prescriptions outside ULSM, or may be in clinical situations where medication is not recommended, or its benefit is uncertain. Moreover, some limitations have been highlighted for LVEF-based classification, e.g., it does not consider the pathophysiological mechanism and specific etiology underlying HF, and there is also variability among the different imaging techniques used to assess LVEF and its measurement is based on technical geometrical assumptions [46,47,48]. Despite these limitations, our results contribute to the understanding and characterization of HF subtypes, and reflect the growing burden of HFpEF.

In conclusion, we provide 20-year evidence that heart failure is a major public health concern, with a high prevalence in the elderly with comorbidities, namely diabetes and CKD. Strategies for the prevention and early treatment of these comorbidities are needed, and could have a huge impact on HF progression and prognosis. In this cohort, considering a contemporary definition, the prevalence of HF was 2.1%, with HFrEF accounting for 16.3% of cases. Our study also revealed a high prevalence of patients with HFpEF (65.4%), raising awareness for the increasing prevalence of this entity in cardiological practice. This finding may also highlight the impact of the lack of effective therapeutic indications in the current guidelines for the HFpEF and HFmrEF, and the potential importance of SGLT2i in reducing the risk of major HF outcomes [38,39]. These results may guide local and national health policies and strategies for HF diagnosis and management.

## Figures and Tables

**Table 1:** Demographics, comorbidities, cardiovascular medications, clinical assessment, and echocardiogram results of the heart failure patients.

**Demographics**	***n***	**%**
Patients (N)	2700	
Age (mean, SD)	74.0	12.1
Females (*n*, %)	1394	51.6
**General Comorbidities**	***n***	**%**
Type 2 diabetes mellitus	1207	44.7
Myocardial infarction	836	31.0
Atrial fibrillation	1110	41.1
Stroke	779	28.9
Peripheral artery disease	199	7.4
Microvascular disease	499	18.5
Chronic kidney disease	1803	66.7
eGFR [45, 60] mL/min	530	19.6
eGFR [30, 45] mL/min	577	21.4
eGFR [15, 30] mL/min	495	18.3
eGFR ≤ 15 mL/min	201	7.4
**Cardiovascular Medication**	***n***	**%**
Hypertension medications	1619	60.0
Low dose acetylsalicylic acid	746	27.6
Receptor P2Y12 antagonists	307	11.4
Statins	1747	64.7
Angiotensin-converting enzyme inhibitors	985	36.5
Angiotensin II receptor blockers	389	14.4
Beta blockers	1620	60.0
High ceiling diuretics	1266	46.9
Aldosterone antagonists	405	15.0
Warfarin	123	4.6
No medication	353	13.1
**Clinical Assessment**	**Median**	**IQR**
Systolic blood pressure (mmHg)	137.0	23.0
Diastolic blood pressure (mmHg)	77.0	15.0
Total body weight (kg)	73.0	19.0
Body mass index (kg/m^2^)	27.7	6.4
Body surface area (m^2^, DuBois D)	1.8	0.3
Waist circumference (cm)	101.0	16.0
**Laboratory Test Results**	**Median**	**IQR**
Hemoglobin (mg/dL)	12.8	2.6
Glycated hemoglobin (%)	5.8	1.1
LDL cholesterol (mg/dL)	106.0	53.0
HDL cholesterol (mg/dL)	43.0	16.0
Total cholesterol (mg/dL)	179.0	59.0
Triglycerides (mg/dL)	111.0	71.0
BNP (pg/mL)	195.7	253.9
Serum creatinine (mg/mL)	1.2	42.3
GFR (mL/min, MDRD)	50.7	83.1
**Echocardiogram Results**	**Median**	**IQR**
Left atrial volume (mL)	42.0	8.0
Left atrial volume, indexed (mL/m^2^)	23.4	4.8
Left atrial diameter (mm)	51.0	8.0
Interventricular septum thickness (mm)	11.0	3.0
Posterior ventricular wall thickness (mm)	9.0	1.0
Left ventricular ejection fraction (%)	59.0	14.0
Left ventricular mass (g)	147.7	48.3
Left ventricular mass indexed (g/m^2^)	83.1	23.1
Interventricular septum thickness (mm)	11.0	3.0

eGFR, estimated glomerular filtration rate; MDRD, Modification of Diet in Renal Disease; BNP, B-type natriuretic peptide; LDL, low-density lipoprotein; HDL, high-density lipoprotein; IRQ, interquartile range.

**Table 2:** Comorbidities and cardiovascular medications of the heart failure patients per subtype.

	HFrEF *n* = 429		HFnrEF *n* = 2199		*p*
**Age (Mean, SD)**	67.0	12.0	74.7	12.0	<0.001
Females (*n*, %)	139	33.9	1209	55.0	<0.001
**General Comorbidities**	***n***	**%**	***n***	**%**	
Type 2 diabetes mellitus	204	46.5	969	44.1	0.421
Myocardial infarction	200	45.6	624	28.4	<0.001
Atrial fibrillation	177	40.3	871	39.6	0.759
Stroke	112	25.5	652	29.6	0.059
Peripheral artery disease	44	10.0	152	6.9	0.050
Microvascular disease	85	19.4	398	18.1	0.500
Chronic kidney disease	297	67.7	1483	67.4	1.000
eGFR [45, 60] mL/min	83	18.9	447	20.3	0.146
eGFR [30, 45] mL/min	105	23.9	462	21.0	
eGFR [15, 30] mL/min	85	19.4	403	18.3	
eGFR ≤ 15 mL/min	24	5.5	171	7.8	
**Cardiovascular Medications**	***n***	**%**	***n***	**%**	***p***
Hypertension medications	316	72.0	1273	57.9	<0.001
Low dose acetylsalicylic acid	167	38.0	574	26.1	<0.001
Receptor P2Y12 antagonists	71	16.2	233	10.6	<0.001
Statins	322	73.3	1386	63.0	<0.001
Angiotensin-converting enzyme inhibitors	244	55.6	719	32.7	<0.001
Angiotensin II receptor blockers	65	14.8	314	14.3	0.816
Beta blockers	348	79.3	1227	55.8	<0.001
High ceiling diuretics	248	56.5	979	44.5	<0.001
Aldosterone antagonists	214	48.7	182	8.3	<0.001
Warfarin	26	5.9	92	4.2	0.171
No medication	35	8.0	314	14.3	<0.001

eGFR, estimated glomerular filtration rate.

**Table 3:** Clinical assessment, laboratory test and echocardiogram results, and diabetes medication of patients with heart failure and type 2 diabetes mellitus.

Clinical Assessment	Median	IQR
Systolic blood pressure (mmHg)	138.0	24.0
Diastolic blood pressure (mmHg)	76.0	15.0
Total body weight (kg)	75.0	19.0
Body mass index (kg/m^2^)	28.4	6.6
Body surface area (m^2^, DuBois D)	1.8	0.2
Waist circumference(cm)	103.0	16.0
**Diabetes Medications**	***n***	**%**
Insulins	323	26.8
Metformin	585	48.5
SGLT-2 inhibitors	105	8.7
DPP-4 inhibitors	501	41.5
Sulfonylurea	118	9.8
GLP-1 receptor agonists	21	1.7
No medication	343	28.4
**Lab Results**	**Median**	**IQR**
Hemoglobin (mg/dL)	12.5	2.6
Glycated hemoglobin (%)	6.6	1.6
LDL cholesterol (mg/dL)	104.0	53.0
HDL cholesterol (mg/dL)	40.0	15.0
Total cholesterol (mg/dL)	170.9	57.5
Triglycerides (mg/dL)	125.0	80.0
BNP (pg/mL)	202.9	304.5
Serum creatinine (mg/mL)	1.5	40.1
GFR (mL/min, MDRD)	38.1	71.9
**Echocardiogram Results**	**Median**	**IQR**
Left atrial volume (mL)	42.0	8.0
Left atrial volume, indexed (mL/m^2^)	23.6	4.8
Left atrial diameter (mm)	51.0	8.0
Interventricular septum thickness (mm)	11.0	3.0
Posterior ventricular wall thickness (mm)	10.0	1.0
Left ventricular ejection fraction (%)	58.0	14.0
Left ventricular mass (g)	153.0	48.3
Left ventricular mass indexed (g/m^2^)	85.2	23.1

eGFR, estimated glomerular filtration rate; MDRD, Modification of Diet in Renal Disease; BNP, B-type natriuretic peptide; LDL, low-density lipoprotein; HDL, high-density lipoprotein; GLP-1 receptor agonists, glucagon-like peptide-1 receptor agonists; SGLT-2 inhibitors, sodium-glucose cotransporter-2 inhibitors; DPP-4i inhibitors, dipeptidyl peptidase 4 inhibitors; IRQ, interquartile range.

**Table 4:** Comorbidities and cardiovascular medications of heart failure patients with and without type 2 diabetes mellitus.

	T2D *n* = 1207		Non-T2D *n* = 1493		*p*
**Age (Mean, SD)**	75.3	10.2	73.0	13.4	<0.001
Females (*n*, %)	617	51.1	777	52.0	0.912
**General Comorbidities**	***n***	**%**	***n***	**%**	***p***
Myocardial infarction	390	32.3	446	29.9	0.263
Atrial fibrillation	517	42.8	593	39.7	0.116
Stroke	395	32.7	384	25.7	<0.001
Peripheral artery disease	119	9.9	80	5.4	<0.001
Microvascular disease	427	35.4	72	4.8	<0.001
Chronic kidney disease	944	78.2	836	56.0	<0.001
eGFR [45, 60] mL/min	208	17.2	322	21.6	<0.001
eGFR [30, 45] mL/min	302	25.0	265	17.7	
eGFR [15, 30] mL/min	288	23.9	200	13.4	
eGFR ≤ 15 mL/min	146	12.1	49	3.3	
**Cardiovascular Medications**	***n***	**%**	***n***	**%**	***p***
Hypertension medications	757	62.7	862	57.7	0.013
Low dose acetylsalicylic acid	377	31.2	369	24.7	<0.001
Receptor P2Y12 antagonists	153	12.7	154	10.3	0.080
Statins	840	69.6	907	60.8	<0.001
Angiotensin-converting enzyme inhibitors	458	37.9	527	35.3	0.165
Angiotensin II receptor blockers	190	15.7	199	13.3	0.087
Beta blockers	767	63.5	853	57.1	<0.001
High ceiling diuretics	659	54.6	607	40.7	<0.001
Aldosterone antagonists	198	16.4	207	13.9	0.051
Warfarin	46	3.8	77	5.2	0.058
No medication	115	9.5	238	15.9	<0.001

eGFR, estimated glomerular filtration rate; IRQ, interquartile range; T2D, type 2 diabetes mellitus.
